# The Replier Replied to, and the Reviewer Reviewed; a Letter to Lunsford P. Yandell, M.D., Containing Strictures and Thoughts on the Errors and False Doctrines of Chemico-Physiology

**Published:** 1844-11

**Authors:** Charles Caldwell


### WESTERN JOURNAL

OF

### MEDICINE AND SURGERY.

NOVEMBER, 1844.

Art. I.—*The Replier replied to, and the Reviewer reviewed*—A letter to Lunsford P. Yandell, M.D., containing Strictures and Thoughts on the errors and false doctrines of Chemico-Physiology. By Charles Caldwell, M.D.

(concluded from page 1.)

Having passed thus hastily over what may be regarded as comparatively minor points, I shall now submit to you a more detailed exposition of the experiments of Fordyce, Banks, and Blagden.

The object of those three philosophers was to ascertain, by experiment, the effect produced on the human body, by a temporary exposure to a very elevated temperature. And that the result of the trial might be the more accurate and satisfactory—and therefore the more valuable, they determined to experiment on their own persons.

For the accomplishment of their purpose, they prepared a suit of three rooms, each from 15 to 20 feet square, and of a corresponding height.

The atmospheres of those apartments they heated to different degrees, ranging, I think, at different times, from 100° to 264° of Fahrenheit. To these varying temperatures did they expose their naked bodies (during periods sufficient to make the trials fair) sometimes individually, and at other times in company with each other—determined, that by diversifying their experiments, under the best concerted plan of action and observation, no ground should be left for cavil or doubt, as to the truth and accuracy of the processes and their results. On no occasion, however, did their persons, even when acted upon by the hottest atmosphere, manifest a temperature higher than from 100° to 100½°, and 101° of Fahrenheit.

At that temperature did they steadily remain, even when the heat of the atmosphere around them, and acting on them, varied from 224° to 264°; and when eggs immersed in cold water and exposed to it were *boiled to hardness* in 20 minutes; and beef-steaks sufficiently *broiled* in it in 13 minutes. No painful or unpleasant sensations moreover were produced on the skin, lips, or eyes, by the portion of the highly heated air, which came into immediate contact with them; nor on the mouth, tongue, fauces, nares, larynx, trachea, or lungs, by the portion which was breathed. Nor was this all.

Not only did the burning atmosphere of the rooms fail to elevate the temperature of the bodies of the experimenters; their bodies rapidly and materially lowered its temperature.

In proof of this; into one of the rooms, at the temperature of 210°, Dr. Blagden entering alone, the thermometer in it soon fell to 198°. The temperature was again raised to 211°; when, on the entrance of Sir Joseph Banks alone, it fell again, in a few minutes, to 198°. And, on the entrance of the three gentlemen at once, the descent of the quicksilver was much more rapid, and, consequently, much greater, in a given period. It is worthy of remark, that the more vigorous the *constitution* of the individual (by which is meant his *vital force and action*) the more rapidly did he reduce the temperature of the atmosphere, and the more slowly and to the less extent was the temperature of his own body elevated. This fact clearly bespeaks the issue to have been the product, not of *chemical*, but of *vital* influence. The vigor of that influence counteracted and held in check the tendency of the heated air of the apartment to raise the temperature of the bodies of the experimenters. But, according to your and Liebig's hypothesis, the reverse should have been the case. The greater the vigor of the constitution and its vital action, the greater is the amount of the oxygen, hydrogen, and carbon consumed, in a given time, and consequently the more intense is the burning. And this burning from within must necessarily co-operate with the heated air without, in the elevation of the personal temperature. When applied to the present case, therefore, the hypothesis you would sustain destroys itself. A strong constitution resisted the heated atmosphere, *because* it was strong; while a feeble one yielded to it, *because* it was *feeble.* This fact, therefore, is fatal to the notion of Liebig—as far at least as a single fact may avail.

Meantime, though the temperature of the experimenters was so slightly increased, their pulses rose in frequency, from their natural standard (say 75 to 80) to 150 beats in a minute. And, of course, the frequency of their respiration was also augmented, as well as the quantity of air which they breathed in a given time.

Such are some of the leading and most significant facts and their accompaniments of the case. Nor is their signification either obscure or equivocal. Far otherwise. It announces in language too clear and definite to be misunderstood, and too emphatic and forcible to be resisted, that, by a process or mode of action, utterly inexplicable on chemical principles, the living human body can and does abstract sensible caloric from the air around it, materially reducing the atmospheric temperature, while, by throwing into a latent condition the matter of heat, thus abstracted, it prevents the increase of its own temperature.

That the human body does thus withdraw caloric from the atmosphere which encircles it, and cool that, without becoming itself correspondingly heated, is a position which you cannot deny; because the facts of the case in question directly and conclusively testify to its truth. But you contend that, in so doing, it acts on principles, and by a process essentially chemical; and that process you attempt to expound. By a few strictures however on your attempt, and on the principles and means on which you depend for its accomplishment, its entire insufficiency may be satisfactorily evinced, and its failure made plain.

In your effort to explain and remove the difficulties that press, like an incubus, on Liebig and his retainers, you say, that

"The effect" (manifested in the preceding experiments) "which strikes one as so wonderful, depends, first, upon the air's being a bad conductor of caloric, and secondly, upon the evaporation of the perspirable matter from the body."

And there, your resources of explanation being exhausted, no further effort to that effect is made by you. If I can show therefore, that in this death-struggle to maintain yourself and your hypothesis, you not only fail to accomplish your purpose, but involve yourself in further entanglements and inconsistencies, and thus render your cause more hopeless, and your prospects more gloomy—should I succeed in doing this, though I shall not presume to tender to you any advice in the premises, nothing forbids me respectfully to submit to you whether it may not be discreet in you to retire from the contest, and allow Professor Liebig hereafter to be his own champion, and to fight his own battles? Under existing circumstances, you have nothing of scientific standing or reputation to gain; though, as relates to both, you may lose not a little. Should you, in the conflict, prove victorious, the victory will redound to the honor of Liebig; because you make battle with his principles, in his name, and under his banner. But, in case of defeat, the disaster and disrepute will fall exclusively on your own head; because, by your fellow-members of the school of Giessen, the catastrophy will be ascribed, not to any failing or fault which attaches to either Liebig, his doctrine, or themselves; but to some defect in your qualifications or conduct—in your prowess—your science—your skill in tactics—your judgment in the selection of weapons—or your dexterity in the use of them. These hints are not actually *offered* to you—especially they are not *pressed* on you—they are merely deposited where you may find them. Employ them as you may think proper, or let them remain unemployed.

In pronouncing atmospherical air 'a bad conductor of caloric,' your object I presume is, to endeavor to make it appear, that it did not readily impart that substance to the persons of Fordyce, Banks, and Blagden, and therefore failed almost entirely to heat them.

If this be the sentiment you mean to convey, permit me to observe, that it is altogether groundless. The air did impart its caloric to the persons of the experimenters very readily and copiously; because, while they were within the atmosphere of the rooms, prepared for their inquiry, its temperature fell both rapidly and extensively. When but a single individual was in contact with it, the whole volume of the air contained in the apartment lost, in a few minutes, in one case, 12° of heat, and in another 13°.

Wherefore did this fall of temperature take place? The answer is plain and not to be controverted. The air parted from its caloric, and communicated it to the body of the gentleman who was in the midst of it. Yet, by such communication, the temperature of his body was not raised. Of this again the cause is obvious. By some form of vital influence, not yet discovered, his body reduced to a latent state the caloric it received from the surrounding air. To such a state the caloric unquestionably *was* reduced, because its influence was no longer experienced by either the atmosphere or the bodies of the experimenters.

The truth of this even you yourself substantially acknowledge; though you do not appear to be conscious of the fact. The following are your words:

"The stratum of air in contact with them" (the experimenters) "*parted with a portion of its caloric*, grew heavier in consequence, and subsided along the floor, making way for a fresh volume of hot air, in its turn to be cooled down, and settle towards the bottom."

Though unwilling to find fault with style and manner in the composition of a scientific paper, I am notwithstanding compelled to pronounce this a very labored and obscure sentence. Owing to either its entanglement and want of lucidity, or to my want of ready perceptiveness, to derive from it any clear and definite idea of the view or conception, under which you wrote it, is beyond my ability. Nor do I hesitate further to pronounce obscurity of expression a natural result of obscurity of thought. It is moreover the result of that *alone*, when its author is deliberate and careful in writing, and has a ready and felicitous command of speech. Now such command is known to be yours, in the literal and full signification of the terms I have employed in expressing it. In plainer and more definite language, and without the slightest disposition to compliment or flatter you, your store of words is unusually abundant; and you draw on it with a corresponding facility and promptness.

I am therefore convinced that, when you penned the paragraph now before me, your mind was puzzled and embarrassed from a barrenness or confusion of ideas—you labored in vain in quest of suitable and satisfactory matter—and your thoughts were misty, vague, and indefinite. Had not this been the case, your phraseology would have been, as it very rarely fails to be, clearly intelligible to me. But haplessly it is not so. Allow me therefore to hope, that you will neither be surprised, nor deem me in a high degree faulty, should I fail in my interpretation of it.

Through the twilight of your expression, however, I am so fortunate as to discry, with sufficient clearness, one act of your mind, which is important to my purpose—your admission of the fact, that the air, in contact with the persons of the experimenters, imparted to them not a scanty, but an abundant portion of its caloric; and that it did so with a rapidity and a readiness far beyond what, in your language already quoted, you seem willing to admit.

In confirmation of the correctness of this interpretation of your meaning, you acknowledge that the air of the rooms in which they experimented, flowed to the persons of the experimenters, in an uninterrupted current, came into contact with them, was immediately cooled, to the extent, in one case, of more than 150° and then, descending their bodies, 'ran along the floor,' in a cool stream or sheet, nobody knows how far, to what point, or for what reason. On neither of these points does either your narrative or philosophy sufficiently inform us.

Though you do not yourself say, that the portion of air, which came into contact with the persons of the experimenters, lost by the act more than 150° of its caloric; yet such was the fact, as the record of the experimenters transmitted to us testifies.

The temperature of the persons of the experimenters was about 100°; while that of the atmosphere at a distance was from 224° to 264°. But when that high-heated atmosphere flowed toward the experimenters, and came into contact with them, it was speedily cooled down to their own temperature of 100°, and, in consequence of its newly acquired weight, descended their persons, as you assert, in a stream or sheet, and thus ran from them, to give place to such other sheets as might succeed it. During this change of place and condition, it was deprived of at least 124° of sensible heat, which, in some way attached to the bodies of the experimenters, without raising their temperature. Here are three or four important facts to be held in remembrance—sensible caloric was withdrawn from the atmosphere, which reduced its temperature—and it attached itself to the bodies of the experimenters, without increasing theirs. Nor was it returned into the atmosphere in a sensible condition. In truth, as will presently appear, it was not returned into the atmosphere at all—but remained insensible and inoperative in the persons of the philosophers.

Thus stands the case, according to your own telling. From what source, then, permit me to inquire, do you derive your authority for alleging, that the atmosphere around them withheld its caloric from the persons of the three philosophers? The record handed down to us gives you no such authority. Nor is it furnished to you by the philosophy of the case, nor by any nor all of the circumstances connected with it.

We are told, in the biography of that distinguished orator, that Sheridan once, sarcastically and with no little effect, charged on an opponent in debate, that for his *wit* he always resorted to his *memory*, and to his *imagination* for his *facts*.

Now though I do not impute to you the practice of a similar expedient, yet, were I to do so, you might find it, in the case before us, no trivial task to free yourself from the charge—very especially from the latter member of it. In the emptiness of your memory, you really seem to have appealed, for facts, to the plenitude of your imagination.

But I am not yet done with your allegation, that atmospherical air, being a bad conductor of caloric, withholds that substance from bodies immersed in it. Notwithstanding what you have said, you will not deny, that the atmosphere of the experiment-room parted from its caloric freely, and supplied it very abundantly to the beef-steaks which it *broiled* in 13 minutes, and to the cold water which it heated sufficiently to cause eggs to be boiled to hardness in it, in 20 minutes. Wherefore then could it not impart the same article, with the same readiness, and to the same amount, to the bodies of the experimenters? Were both of them lifeless, is not the flesh of a man as receptive of caloric as the flesh of an ox? You will not, I persuade myself, deny that it is. From what cause then did the air fail to impart its caloric to living human flesh, as easily and copiously as to dead ox-flesh? To the ground or rather source of this question your review makes no reference. Yet are you bound to refer to it, and to answer satisfactorily the question itself, or frankly acknowledge that it constitutes an objection fatal to your hypothesis. The dilemma is thus prepared and presented. Nor does any privilege remain to you, save the choice of the horn on which you are to hang. I shall only add, in this place, that the very fact, that the atmosphere of the room raised the mercury in the thermometer, (*through glass*, which is a bad conductor of heat) to the 264°th, on the scale of Fahrenheit, is of itself a conclusive proof of the facility with which it parted from caloric. And it constitutes, to your attempted defence of your hypothesis, another objection, whose subversion is utterly and even hopelessly impracticable.

As you have entirely failed then, to explain, by your first resource, the 'effect' which you acknowledge 'strikes one as so wonderful,' I shall now analyze your second, in order to ascertain whether it is in any measure better fitted to solve the question, and remove the difficulty. It is the process of *evaporation*, which you deem so powerful and efficient, as to be able to control and modify, at pleasure, every form and degree of the heat-producing function. But, if I mistake not greatly, you will find that, in the instance I am now considering, you have far overrated the power and efficiency of that boasted agent.

Antecedently, however, to my special consideration of the process, I feel authorized to observe, as a general and leading remark, that, in the case now before us, evaporation is a two-edged sword, which cuts behind, as well as before—*against* your hypothesis, as effectually as *for* it. Both the beef-steaks that were broiled, and the water, in which the eggs were boiled, received of necessity a high degree of heat. Yet did they both *very copiously exhale* from their surfaces—more copiously perhaps than any equal extent of surface of the bodies of the experimenters. Wherefore then did not the temperature of the latter substances rise to as great a height, as that of the former? In a mere chemical point of view, exhalation from one surface ought to carry along with it as much caloric, as exhalation from another. Nor will you be likely to deny that it does so. One of the difficulties then which you are called on to encounter, but not the only one, is involved in this question, why did it act differently in the case I am considering? But, stubborn as this difficulty is likely to prove, obstacles to your hypothesis of a more intractable character are yet to be presented to you.

When you shall have scrutinized the subject with the requisite degree of severity and thoroughness, (which from sundry considerations I am satisfied you have never yet done), you will find that, for the production of the effects you exact from it, the labors of your favorite agent *exhalation* must be herculean. And you will further find, that, in providing the materials on which to found your estimate of its power, you will be again compelled to draw on supposition and inventiveness much more largely than on observation and memory. In still plainer and more discouraging language, you will be obliged to rely on the two former sources alone, in as much as the two latter can furnish you with nothing suited to your purpose. For your whole theory is an imaginary fabric.

To begin our scrutiny of this matter, with the eyes of the three experimenters. As far as you are informed on the subject, they were neither burnt, nor in any way annoyed by the hot air in contact with them. You are compelled therefore to believe that they experienced from that agent neither pain nor discomfort. But you have no reason to imagine that they were protected from injury by an augmented exhalation from them; because you are told of the existence of no such augmentation. As a philosopher therefore, and an accurate inquirer after truth, you are forbidden to fabricate or conjecture its existence. Here then was the immunity of an important, delicate, and susceptible organ without any known increase of evaporation.

You are not told that the air inspired by the experimenters, though it broiled beef-steaks, and boiled eggs, pained or irritated, by its burning temperature, either the lips, mouth, or fauces, the nares, the larynx, the bronchiæ, or any other portion of the respiratory apparatus. Hence you are constrained to believe that no such discomfortable effects were produced by it. Yet no report has given you reason to allege, that exhalation from those organs was in any measure augmented. Whatever therefore may be your secret wish or suspicion on the subject, you will not, I am sure, either discredit your philosophy, or blemish your love of truth, by *asserting* its augmentation.

Nor, when the experimenters assure you, as they do, that, instead of *exhaling* and *gas-forming*, their persons were converted into *condensing* and *water-forming* bodies, can you fail to believe them. For not only were they men of high and well-trained intellects, which qualified them to discern and judge; they were surpassed by none of their contemporaries, in rectitude, and moral standing. Their report therefore must be received as correct and indubitable. And that report is no less than a death-blow to your *exhalation* hypothesis.

The three philosophers tell you, that their persons, by their comparative coolness, attracted from the vapor in the atmosphere around them, so much caloric, as to condense that vapor into water. And they further tell you, that the water thus formed was so abundant, as to settle on their skins, in a statum or sheet, and even to trickle down them in minute streams. To prove to you moreover that this *was water* formed from the atmosphere, and not sweat, they state, as an additional fact, that, on the surface of a Florence flask held in their hands, at the temperature of 120°, the condensed uapor settled in the form of water; and that, when one covering of that fluid was wiped off the glass vessel, by a napkin, another was soon condensed from the same source, and deposited in its place.

Permit me here to put to you another question. Do you, can you, seriously and sincerely believe, that while water was thus forming and settling on the surfaces of the bodies of the experimenters, by their inhalation of *caloric from without*, perspirable matter was *exhaling* from them, by the action of the *same substance on* them or *within them?* In other words; can the human skin be, at the same moment, and in the same spot, at once an imbibing and condensing, and an eliminating and dissipating surface? Can it, at the same moment, and on the same spot, act in two contrary modes, and in two exactly opposite directions?

We are told, I believe correctly, (indeed you have told us yourself) that, in a humid atmosphere, the evaporation of the perspirable matter is greatly diminished, if not entirely prevented; and that hence arises the oppressive influence exercised on us by air, at once inordinately heated and surcharged with moisture. And, that from the same source arises the same oppressiveness of a long continuance in an over-heated water or vapor bath. If then evaporation be impeded or completely arrested, by a humid atmosphere alone, how can that process be performed, under the combined action and opposition of a moist and heated atmosphere, and a sheet of water on the skin itself? Is it possible for the surface of the body, which condenses into water the atmospherical vapor in contact with it, to secrete itself an aqueous vapor out of the blood, and issue it into the atmosphere? Can it, at the same moment, perform two processes, or forms of action, so immensely different from each other? *Never*—I repeat confidently—NEVER. And, if I mistake not immensely, truth and nature, by way of their sanction, echo the reply.

Individually I am unable, after the strictest examination of the subject, to perceive any ground of belief, that the entire body of man can, at the same time, abstract and imbibe caloric from surrounding atmospheric vapor, and discharge it into the atmosphere by the evaporation of perspirable matter. As well may you contend that two opposite currents of any other substance, whether ponderable or imponderable, may meet and pass directly through each other, without mutual collision and interruption. Condensation and evaporation are two contrary processes, produced on contrary principles, and by contrary modes of action. Your notion, therefore, that they may coexist on the same surface, is as unphilosophical, not to say preposterous, as that which would contend for the harmonious coexistence, in the same spot, of any other two contraries.

Nor is this all. According to your own view of the matter, a much larger amount of caloric must have entered the bodies of the experimenters from without, than was produced within them, by their own action, and carried away by exhalation from their surfaces. Under these circumstances, unless they had possessed a peculiar power to neutralize caloric, and render it insensible, their temperature must have been increased by it.

But were I even to concede the point, that the evaporation of their perspirable matter did prevent the temperature of the bodies of the experimenters from rising; had that evaporation from the person of *one* of them such an amount of cooling power, as to reduce, in a few minutes, to the extent of 13°, the temperature of the whole atmosphere of a room of no inconsiderable dimensions? To this question you will not hazard an affirmative answer. Were you to do so, the act would indicate in you a want no less of sound science than of sound judgment, to an extent far beyond what I am willing to admit. In truth, I perceive not a single principle or mode of action, on or by which evaporation of perspirable matter from the body of an individual can, to any extent, *or even at all*, cool any portion of the atmosphere around him. To another and very different cause, therefore, must the *frigorific* agency referred to be ascribed.

Nor is that cause either deeply or darkly concealed from an enlightened and unprejudiced inquirer. It is the readiness with which the air of the apartment gave up its caloric, and communicated it to the persons of the experimenters, who came into contact with it. To no cause other than such surrender of its caloric by the atmosphere, and the communication of it to the body of the individual who entered the room, can the sudden reduction of temperature be ascribed. Nor will you deny that, all things considered, the reduction was great as well as sudden.

The very conception that the exhalation of perspirable matter from the body of the experimenter in the room exerted this frigorific influence, you will excuse me I hope for pronouncing an act of utter *thoughtlessness*, and want of reflection. For offering this remark I have two reasons, either of which is to myself satisfactory.

Had the exhalation existed, it would, as already remarked, have been altogether incompetent to the production of the effect. But that it did not exist, there is strong reason to believe. Let me here be distinctly and perfectly understood. My meaning is, that when the temperature of the atmosphere of the room was at its height (from 224° to 264°) we have no satisfactory reason to believe that the experimenter who subjected himself to its action, was thrown by it into a copious sweat, and exhalation. On the contrary we have very probable ground of belief that he was not.

In the first place, when the temperature of the atmosphere of the room ranged from 100° to 120°, we are given to understand that the experimenter exposed to it perspired and exhaled abundantly. But when the temperature was raised to 200°, and upward, we are informed, not that the person exposed to its influence sweated and exhaled, but that his body condensed into water the vapor already contained in the atmosphere. And had he copiously perspired and exhaled at the same time, the fact would have been recorded, (if for no other reason) on account of its *singularity*—to say nothing of its significance and weight, in the settlement of the question under discussion. Nor is this all.

Though I do not assert it to be a *fact*, yet do I regard it as a *strong probability*, that air of a very high temperature

(say from 150° to 200° and upward of Fahrenheit) acting on the human skin, instead of *promoting* perspiration, actually *prevents it.* Perspiration is a natural and healthy process, to be promoted only by natural and salutary impressions. But, the impression made on the skin, by a very high temperature is *deleterious*—it amounts to *scorching*—or something near to it—under which condition perspiration does not occur. The *vis conservatrix* (a power which unquestionably exists) resorts, for the preservation of the system, to some other mode of action, adapted to the occasion.

Stimulate any organ of the body up to the point of *disagreeable* irritation, which is tantamount to disease, and you greatly impair its natural and healthy function—if you do not for the time, entirely suspend it. Plunge your feet and legs into water so hot that it burns and gives pain—and no perspiration is produced. Scorch your hands or face, by approximating them too closely to a hot fire, and the same will be true, in relation to them. They exhale nothing, but remain dry and rather harsh. The cutaneous vessels become too deeply gorged with blood, and are thereby too much altered in their condition to secrete from the fluid which overdistends them the matter of perspiration.

Nor do I believe the case to be different with the cutaneous vessels, when they are irritated by atmospherical air, at the temperature of 200° and upwards. Their healthy condition is so essentially deranged, that they cannot perform their *natural* function. But that function is the secretion of the perspirable fluid, which, in the case before us, is temporarily suspended—suspended I mean until the removal of irritation and its efficient cause.

In further evidence of the correctness of my view on this subject, take two patients in the acme of the hot-stage of an intermittent—spunge with cold water the person of one of them, allowing the other to remain untouched; and mark the issue. You will find that in the former, not only will the febrile heat be soonest subdued—*the sweating stage will be soonest induced.* The reason is plain. The temperature of the skin, which had been previously too high, is reduced by the sponging process to the 'sweating point.' For the same reason (the reduction of temperature) venesection, judiciously employed, promotes both *sweating* and *purging;* to the *latter* of which processes secretion is as essential as to the *former.* In these cases you, *as a chemist*, make the effect *antecede* and *control* the cause, instead of following and obeying it. But nature tolerates no such perversion.

Again. At the time of the performance of the experiments by Fordyce, Banks, and Blagden, the question was keenly agitated, whether the immunity of those gentlemen from burning, by the highly heated air, arose from *chemical* or *physiological* influences? And the experimenters themselves, who were well informed in both the branches of science referred to, declared unanimously in favor of the latter. They persistently asserted, that chemistry had no agency in their protection. And, in justice to them, as well as to the cause of truth, I do not hesitate to express my persuasion, that they were fully as well (or rather much better) prepared to judge of and interpret their own experiments, than either you, Liebig, or myself—or than all of us united.

At the period here alluded to *Black's* theory of animal heat was as strenuously opposed and defended as is *Liebig's* theory at present. But where is the *former* theory *now?* Precisely where the *latter will* be, before the lapse of the *tithe* of a century—peacefully reposing under the waters of forgetfulness—or remembered only as a signal display of deep error and bold pretension in its author, and of corresponding credulity and delusion in its adopters and advocates.

On the whole, then, as a fair and legitimate inference from the foregoing chain of facts and argument, I feel authorized to say, and do not hesitate to say positively, that one of the two following positions is true:—Either the experiments of Fordyce, Banks, and Blagden, and their extraordinary issues are incorrectly reported, or the living human body possesses a power hitherto unexplained, of protecting itself from the deleterious influence of an overheated atmosphere, by changing very abundantly *sensible* into *latent* caloric. And let me ask you to bear in mind, and calmly reflect on what I have said, respecting the influence of a very excessive temperature on the human skin—that it seems to *prevent* it from *secreting*, and of course from *exhaling* perspirable matter. If this be true, it settles completely the interpretation of the experiments, and rescues them forever from chemical ambition, usurpation, and perversion. But, to proceed with my remarks.

Your singular comment on the following passage, extracted from my Critique, deserves perhaps a succinct notice.

"Take," I observe, "a large tubful of water heated to the temperature of 120° of Fahrenheit. Immerse your feet and legs in it, and the sense of burning produced by it will be painful to you. Allow your limbs to be still for a few minutes, and the burning will cease. Remove them to another place in the water several inches distant, and the burning will be reproduced. Hold them again motionless, and again you will be freed from pain."

With the position stated in this quotation, you make short work. The following are your words. Of the sentiment you designed to express by them I am not very confident. And the reason of my uncertainty may appear singular to you. The opinion which your language seems best calculated to convey, is so widely apart from what I regard as either *fact* or *probability*, that, notwithstanding the extent of your mistakes elsewhere, I am half inclined to doubt, whether, in the present case, I may not impute to you a notion you did not mean to broach.

"The cause of this" (the aggregate of the phenomena represented in the foregoing extract from my Critique) "is so palpable, that it must have occurred to the mind of the *casual reader*. It is manifestly due to the circulation of the blood in the limb. The blood being 100° reduces the temperature of the water, ('a large tubful!!') and is *itself heated*, at the same time; but as the quantity heated is small in proportion to the mass of that fluid, the general temperature is not sensibly raised in the interval during which the experiment is continued, and *might not be increased at all;* the perspiratory process and consequent evaporation carrying off the excess of calorific."

Instead of applying to this extraordinary paragraph, with a view to the designation of its character *in words*, any of the condemnatory epithets, which it invites, I shall proceed at once to a brief analysis of it, *show* what it is *in fact*, and leave to others the task of bestowing on it a suitable name. And I feel persuaded, that, in the baptismal ceremony, no predilection for an authoritative appellative will be likely to tempt a judicious sponsor to affix to it, as a *soubriquet*, the name of either Black or Priestley, Lavoisier or Davy.

Permit me then to begin my analysis, by putting to you a few questions, plain, indeed, and unceremonious, but perhaps sufficiently significant and searching.

How do you know that the blood in the limb immersed in warm water 'is itself heated,' and that by that means the water, for some distance around the limb, is cooled? Have you ever either witnessed a decisive experiment on the subject, or made one yourself? Have you, for example, drawn blood from the immersed limb, ascertained its temperature, and compared it with the temperature of the blood extracted from other parts of the body? Or have you taken blood from the same limb before and after immersion, and ascertained the temperature of that drawn at the latter period to be the highest?

If you have performed any or all of these experiments, with such others as may be requisite to settle the question, and have found the blood in the immersed limb to be always the *warmest*, then are you privileged to state your knowledge and experience to that effect; and your testimony will be deemed valid and valuable. But, if you have not thus experimented and thus discovered (and I verily believe you have done neither) on what ground, or for what purpose have you first asserted that the temperature of the blood in the immersed limb is increased, and then, by a semi-condemnatory, or self-contradictory equivoque, acknowledge that it 'might not be increased?'—in other and more significant terms, that you did not know whether it was or not—but that you conjectured and hoped it was; because that issue would best com port with the exigency of your hypothesis.

Having proposed these questions, I shall reply to them myself; and, in so doing, I shall express at least a suspicion that you have asserted the increased temperature of the blood, if not perfectly *at random*, at least without being warranted, by sufficient authority, to speak so confidently to that effect. And I therefore further say, that if such be the case, the assertion was not unexceptionable in you; because it involves a departure from the strict requisitions of philosophy, and the principles and rules of sound discussion. Nor is this all. It indicates a disposition to resort, in cases of difficulty, to assertion, as a substitute for fact. It is in reality a pretension to knowledge which you have not yet attained; and which, without accurate experiments not easily performed, you will find it impossible to attain.

Nor have I yet exhausted the whole store of testimony opposed to your hypothesis. The testimony of analogy, as far as it may avail, is directly against it.

The blood of the three experimenters was not increased in temperature more than one and a half or two degrees, by an atmosphere raised to 264° of Fahrenheit. In truth I am not convinced that it was raised at all. The thermometer, employed to ascertain the temperature of these philosophers, was always, I believe, applied under the tongue, or in the axilla. In either place the heated air of the room had free access to it, and was, if not the entire, at least an auxiliary cause of the elevation of the mercury.

If then a temperature of from 224° to 264°, acting on the whole body, did not augment the heat of the blood, it is highly improbable that a temperature of 120°, acting on but a small portion of the lower extremities, increases the temperature of that fluid. Between your *cause* and your *effect* there is an utter want of proportion—the *latter* being far too large for the *former*.

True; in one case, the application of the caloric is made by water, while, in the other, it is made by air. That however cannot much avail you. The difference between the readiness, with which those two substances part from caloric, cannot be so employed by you, as to account, with any semblance of fairness, for the difference you are anxious to make in the result. It cannot enable you to make it appear, by any form of reasoning, or by anything short of positive experiment, that, while, in one case, a temperature of 264° could not augment the temperature of the blood, in another, a temperature of 120° did augment it.

Once more. Are you convinced, by unexceptionable evidence, that water heated to 120°, increases the temperature even of the skin and the other tissues of the immersed leg, including, of course, the coats of the arteries and veins, all of which are superimposed on the blood, as a protection from the water? Does this question surprise you? and are you prepared to answer it promptly and dogmatically in the affirmative? If so; let me hope that you will excuse me for being somewhat reluctant to concur with you in your answer. *I* am *not* convinced that the skin and the immediately subjacent tissues of the immersed leg *are* increased in temperature, above the temperature of the blood in general.

I do not however contend for the reverse of this. My opinion is suspended, and shall so continue, until it shall have attained a fair claim to be *called an opinion.* And such claim it can derive only from authentic evidence. Without such evidence, what the 'million' *call opinion*, is nothing but conjecture. And, in the present case, that is perhaps the proper appellation for both your (so called) opinion and mine. As relates to the matter at issue, neither of us possesses conclusive evidence.

I am probably myself the author of the experiment in question, as a form of trial, to be employed as evidence, in a philosophical inquiry. And I have never performed it in such a way, as to convince myself of the real temperature of the blood of the immersed limb, and that of the tissues whidh surround and protect it. I do not feel authorized, therefore, to speak of it with positiveness. Much less do I consider you authorized to speak positively of it, who, I am inclined to suspect, have never experimented on the question at all. I feel perfectly satisfied that your attempt at an explanation of the phenomenon has no foundation in fact. The error into which you have fallen is more fully exposed, by the following case.

John Hunter took a *living* and a *dead* penis, at the temperature of 92°, and, immersing each in a separate bowl of water, at the temperature of 118°, the *former* rose to 102¼°, and the latter to 114°. And the water containing the living penis was cooled far below that which held the dead one. So great was the difference in this respect, that when the thermometer was placed in the water, by the side of the living penis, the mercury fell to 104°. But, when the instrument was removed from that situation, and immersed in the water, by the side of the dead penis, the mercury rose again to 117°—the water in that bowl having lost but a single degree of its original temperature—while that in the other had lost 14°.

On what ground, except an *occult vital one* will you attempt to explain this striking phenomenon? Pray, do not reply, that the bowlful of water was reduced in temperature 14°, by the small quantity of blood which circulated through the vessels of the living penis—else shall I be compelled to consider you eitheir *in* jest, or *out of* your right mind. Your cause will be again utterly out of proportion to the effect to be explained—I mean that it will be far too feeble for it. And such is also the case with respect to your attempt to account for the cooling of a tubful of hot water, by the immersion in it of the feet and legs. The cause assigned by you I repeat is entirely incompetent to the production of the effect. Nor is there, as far as I can perceive, the slightest conformity of relation between them. And, notwithstanding your allegation, that the explanation assigned by you, for the cooling of hot water, by the immersion of the human feet and legs, is so palpable, that it must have occurred to the '*casual reader*'—notwithstanding this half-boast, half sneer, on the occasion, permit me to offer you an assurance, that I have not yet met with a single 'reader,' whether 'casual' or regular, who does not reject your explanation—highly 'palpable' and satisfactory as you pronounce it. So striking is the difference between the estimation which writers themselves not unfrequently set on their own thoughts and decisions, and that which is set on them, by the bulk of their readers! I am therefore compelled to adhere to my conception on this topic, heretofore expressed, that the living body of man possesses a capability, not yet explained, of converting *sensible* into *latent* heat, its own temperature experiencing, in the process, but little alteration.

Before concluding this letter, permit me to refer, as briefly as may comport with the intelligibility of my remarks, and in other respects suit my purpose, to an effort you have made to defend Liebig, in one of his positions, in which I consider him, and shall endeavor to prove him utterly indefensible. *His* false doctrine, and *your* inefficient effort to expound and sustain it, are as follows:

"All *vital activity*," (says the Professor of Giessen) "arises from the mutual action of the oxygen of the atmosphere and the elements of food."

The doctrine, or rather notion, here unconditionally proclaimed, is sufficiently extraordinary and startling; yet not more so, than your comment on it.

"All this," you say, "is *intelligible* enough."

And so it is, until you rendered it *unintelligible*, by an effort to elucidate or obscure it (I can hardly tell which) by the following self-inconsistent and self-contradictory expressions. But let it be distinctly understood, that while I admit Professor Liebig's proposition to be '*intelligible*,' I am far from admitting that it is also correct. Far otherwise. In your defence of it, you say:

"Activity results from the chemical changes, but a principle is resident in vital beings other than a chemical power—a peculiar force, as expressed by Liebig, because it exhibits manifestations which are found in no other force. This force moulds and shapes the organism, directs muscular motion, and the secretory processes, and supplies to the oxygen inspired from the air the carbon and hydrogen to be consumed in a slow combustion. This principle extinct, or impaired by an injury to the nerves, the fuel is no longer furnished, and the vital temperature declines."

On this extract from your own pen I offer no comment; because I feel my incompetency to the task. I can neither write nor speak about what I am unable to understand. And such is my case in the present instance. So profound is your matter, so dusky and confused your style and manner, or so dull and unimpressible are my perceptive powers, that your meaning is either beyond my comprehension, or in some other way concealed from it.

Through the thick haze, which appears to me to have enveloped you mind, when you composed the extract, I perceive, or fancy I perceive, the outlines and half-developed images of flagrant error, and, as already intimated, self-inconsistency and self-contradiction. But, in the whole mass, there is neither a feature, nor even a line so distinct and well defined, as to be susceptible, by aught that I can do, of either analysis, description, or comment. I therefore leave it, without further notice, to the consideration of others, who may be better prepared to examine and appreciate it. On the assertion, however, of Liebig, that

"*All vital activity* arises from the mutual action of the oxygen of the atmosphere, and the elements of the food:"

let me ask your attention to a few remarks. And, if I am not mistaken, it will require but a few, and those of the plainest and simplest kind, to make it clearly appear to every intelligent and unprejudiced mind, that the German Professor has, in the preceding passage, fallen into an error as gross and glaring, as man can commit, or language express. In direct opposition to the time-worn but never-disputed maxim, '*ex nihilo, nihil fit,*'—out of nothing, nothing can be made—he asserts that out of nothing, *something* can be made. He represents an *absence* or *want* to be equal to a *presence* or *possession*—a positive *vacuum*, to an actual *plenum.* To prove this from his own words:

*Oxygen* is dead matter. So are *carbon, hydrogen,* and *nitrogen,* 'the elements of our food.' Mix, combine, and amalgamate those substances *exclusively* with each other, in any way you may please, or can devise, and they form nothing but a mass of matter *essentially* dead.

This position, *in the abstract,* no one—not even Liebig himself, will venture to deny. Yet does that writer, when he connects it with his hypothesis respecting the source of vitality, positively deny it, in the quotation I am considering. By the 'mutual action' of those four *dead* material elements, he openly declares that *living* matter is produced. For, no where, but in *living matter,* can 'vital activity' exist. To deny this, or to assert the reverse of it, would be an act of preposterous folly. Yet does he, I repeat, in express terms, assert, that 'from the mutual action' of those four *dead substances alone,* '*all vital activity*' (that is all power to act *vitally*—or all *vital* power to act—*vital power,* in fact, in every interpretation that can attach to the expression) '*arises*'*!!* Nor can anything be more *flagrantly contradictory of truth!*

If I am correct in my interpretation of Liebig—if he does assert that, 'from the mutual action' of *dead elements,* 'arises matter possessed of 'vital activity'—and he expressly does so—in such a case it is not possible that you will or can persist in your defence of his false doctrines. Conscience will unite with judgment in impelling you to abandon it, and become the advocate of truth. And, if you perceive me to be incorrect in my version of Liebig's position, I pray you to oblige me, by disclosing to myself privately, or to the world publicly, the mistake I have committed, and I will promptly renounce it. In either case I shall experience sincere gratification. For it is truth and not victory, for which I contend. And now let me close this letter, by asking, once more, the same question, which, without having received a reply, I have heretofore proposed scores of times to yourself, and other members of the chemico-physiological school:

What is the genuine meaning of the phrases—animal chemistry—vegetable chemistry—or vital chemistry?—for I presume the import of the three forms of expression is the same.

Do the advocates of the doctrines of animal and vegetable chemistry contend, that, in the *formation* of living animal and vegetable tissues and secretions, the forces called *chemical affinities* take part, as positive and efficient agents, in like manner as they do, in the production of various sorts of *dead* matter? In the formation, for example, of a leaf, a blossom, or any sort of fruit; and in the formation of any animal fluid, tissue, or organ,—of the blood, the bile, the urine—a muscle, the eye, the kidney, the liver, the pancreas, the brain, or any other kind of organized and living animal or vegetable matter—is it contended that, in the formation of these products, the chemical forces act as they do, in the formation of granite, marble, silicious crystal, sulphuric acid, sulphate of iron, muriate of soda, acetate of lead, sulphate of magnesia, or any other sort of dead matter? Is it in fact contended that, in the former cases, they positively *act at all*, instead of being *acted on*, by a superior and controlling force? Is it contended that they are *agents* and not *subjects?* If such be the doctrine of the animal and vegetable chemists, I believe them to be as deeply and essentially merged in error, as they would be, did they contend that the brick, the mortar, and the trowel, are *spontaneous* and *positive agents*, in the construction of a wall, instead of being subjects and tools in the hands of the mason—that the paint, the pencil, and the palette are *active of themselves* in the process of painting—or that the chissel and the mallet are *agents* and not *subjects* in the art of sculpture.

In architecture, the brick, the mortar, the trowel, the timber, and the edge-tools all possess their natural properties—

their form, their attraction of gravity, their attraction of cohesion, some of them their elasticity, and several other essential attributes. In the erection of the fabric, however, these attributes are not *agents*, but *subjects* expressly under the control of the builder. Of all the materials and instruments of the sculptor, the painter, and the agriculturist, the same is true. They are *subjects* to be acted *on* and *with*—not *agents* to act of themselves. The builders, the painter, the sculptor, and the agriculturist themselves are the vital and directing principle of action of the whole.

Such is my doctrine. And its applicability to the case I am considering is direct and obvious. In the construction of living organized beings, the constituent materials employed in the process probably part from none of the properties they possessed as *dead matter*. They retain their form, their attraction of gravity, their attraction of cohesion, and their chemical attraction. But these attributes take no active and efficient part, in the formation of the living organized fabric. They are but *passive subjects*, not *operative agents*. They are subject to the control of the vital principle, which bears to them the same relation which the wall-builder does to his brick, mortar, and trowel, the painter to his pencil, paint, and palette, and the sculptor to his mallet and chissel. And as soon shall the paint, brush, and palette, without the guiding hand of the artist, produce a picture, or the chissel and mallet a statue, without that of the sculptor, as oxygen, carbon, hydrogen, and nitrogen a living, organized being, without the agency of the vital principle.

As well therefore may you invent and bestow on *wall-building* a name designed to express the fallacious notion, that the brick, mortar, and trowel *perform the work themselves*, or fabricate for painting an appellation intended to set forth the pretension, that the pencil, paint, and palette can, of themselves, execute a picture, as employ, as you do, the deceptive phrases 'animal chemistry,' vegetable chemistry,' and 'vital chemistry.' You would not, by your new-moulded expressions, mislead the unthinking multitude more essentially, than you do, by those already in use. By the latter forms of expression, you produce in many, the groundless belief, that *dead* performs the work of *living* matter; and you could not, by the former, effectuate anything more deeply preposterous.

Another analogical view of the matter may perhaps present it in a more impressive and satisfactory light.

Each private of a skilfully enlisted and well-disciplined army possesses, on the field of battle, all the qualities of a thoroughly trained and competent soldier. He is strong and active, hardy, fearless and brave, and perfectly adroit in military movements and the use of his arms. And all these attributes are *elements* essential to the issue of a well-fought and victorious battle. But they cannot *of themselves* achieve that issue. They are but means to be employed in its achievement by a superior agent. And that agent is the commander-in-chief, who is the *vital principle* of the army. Nor, without him, could the soldiers, with all their high qualities, *as soldiers*, any more accomplish victory, than could the chissel and the hammer complete a statue without the directing hand of the sculptor. The commander produces and governs all the tactical manœuvres of the soldiery, precisely as if they were automatons composed of dead matter—precisely as the chess-player moves the pieces of ivory in the game which he plays. And as soon shall the soldiers, *of themselves*, gain a battle, or the chess-men, a game of chess, as 'the oxygen of the atmosphere, and the 'elements of food' construct or support a living animal, without the agency of the vital principle. Nor, though his hypothesis is a *positive denial of it*, will Liebig, or any of his followers, venture, *in words*, to deny this truth.

Finally; I am anxious that my opinion of Liebig be correctly understood. And it is as follows:

In chemistry and some of its applications, I regard him as one of the great lights and leaders of the age. But, in physiology, pathology, and medicine at large, I consider him as directly the reverse—one of the most wild and reckless *speculators* and *misleaders*, not to say *impostors*, of any age.

Such, in relation to the formation and economy of living organized matter, are the principles and doctrines which I have long adhered to, and endeavored to defend; because I believe them to be founded in truth. And, unless they shall be assailed hereafter, by objections much more powerful than any or all of those that have been hitherto arrayed against them, you are correct in alleging, that I shall not be likely to '*give them up, until I shall have first given up the ghost.*'

With high and sincere regard, I am,
My dear sir,
Very cordially
Yours.
CH. CALDWELL.

June, 1844.

P. S. Though this letter is already considerably longer than I either wished or designed it to be, I must notwithstanding ask the favor of you to indulge me in the addition of a brief postscript.

I have long been, and still am, as already mentioned, exceedingly anxious to receive from you, or some other leading chemico-physiologist, whose opinion may be regarded as *orthodox*, and *authoritative*, a sound definition of '*vital chemistry*,' either *animal* or *vegetable.* My desire is, that the definition may be one so clearly and perfectly intelligible to me, that I may be able to make it the subject of a few remarks, either in approbation or condemnation of it, as my judgment may dictate. And, with a view to facilitate a compliance with my request, I will offer myself, on the *general subject*, two definitions, closely relevant to the end contemplated. A comment on them may, I think, be so moulded by you, as to answer my purpose.

By chemistry I understand that science, which treats of the reciprocal action of the *corpuscles* or *primitive particles* of matter, placed at inappreciable distances from each other. Of this action the solid products are bounded by *right lines* and *angles*, and constitute masses of matter, possessing in their structure, no fitness for the performance of any given sort of operation, or the production of any specific effect.

By physiology I understand that science, which also treats of the mutual action of corpuscles or minute portions of matter, placed at like distances from each other. But of this action the solid products are widely different from the foregoing ones. Instead of right lines and angles, they are bounded by *curved* lines. And, in form, they are *organs* or *instruments* expressly designed and fitted by nature for the performance of given sorts of action, and the production of certain specific effects. They are structural means expressly prepared for ulterior purposes.

Do me the favor to inform me whether you concur with me in these definitions; and oblige me further by stating, what relation they bear to your definition of 'vital chemistry.' And, provided you agree with me, in my definitions, have the goodness to say further, whether you also agree, in opinion, with Professor Liebig, when he asserts that *physiological processes* are performed on principles 'purely chemical?' For, in his Animal Chemistry, such expressly are several of his assertions.

One objection more to your theory of animal heat, and I am done. And, if I mistake not, you will find it both stubborn and formidable.

After an operation for aneurism on a large and principal artery (say the popliteal) but *very little* blood circulates through the limb, especially the lower portion of it, for *several days*, and but a *moderate quantity*, for *several weeks.* Yet does the limb, cold at first, regain, *in a short time*, its *healthy temperature.* This it does when the amount of blood circulating through it is exceedingly small. But the blood alone conveys to it all the oxygen, hydrogen, and carbon which it receives. Those ingredients in it are, of course, correspondingly small in quantity. It contains therefore but very little *fuel* to be consumed in its '*stove*,' and but very little oxygen to consume it.

From what source then does the limb derive the caloric which restores its natural temperature? From the combustion of carbon and hydrogen it cannot derive it; because, I say, it is destitute of those combustibles.

When the surgeon applies his ligatures to the diseased artery, suppose he should also tie up the nerves leading to the limb, so as to prevent their action—or, suppose he should *bisect* each of them in the operation; would the natural temperature then be restored to the member in a short time? No, certainly; it would never be restored.

Hence, therefore, do I feel fully justified in asserting, as a conclusion *demonstratively* certain, that the process, which reproduces the temperature of the limb, while blood is wanting to it, and cerebral influence supplied to it through the nerves, is *vital action*, and not *chemical combustion*.

If you can detect any *unsoundness*, in the foregoing remarks, or in the conclusion deduced from them, your exposure of it will be received by me as a favor. For I am laboring honestly in quest of TRUTH. And any one who will effectually aid me, in my enterprise, will be hailed and regarded by me, as an *instructor* and a *benefactor*.

